# Observation of spin-polarized photoconductivity in (Ga,Mn)As/GaAs heterojunction without magnetic field

**DOI:** 10.1038/srep40558

**Published:** 2017-01-13

**Authors:** Qing Wu, Yu Liu, Hailong Wang, Yuan Li, Wei Huang, Jianhua Zhao, Yonghai Chen

**Affiliations:** 1 Key Laboratory of Semiconductor Materials Science, Beijing Key Laboratory of Low Dimensional Semiconductor Materials and Devices, Institute of Semiconductors, Chinese Academy of Sciences, and University of Chinese Academy of Sciences, Beijing, 100083, China; 2 State Key Laboratory of Superlattices and Microstructures, Institute of Semiconductors, Chinese Academy of Sciences, and University of Chinese Academy of Sciences, Beijing, 100083, China

## Abstract

In the absent of magnetic field, we have observed the anisotropic spin polarization degree of photoconduction (SPD-PC) in (Ga,Mn)As/GaAs heterojunction. We think three kinds of mechanisms contribute to the magnetic related signal, (*i*) (Ga,Mn)As self-producing due to the valence band polarization, (*ii*) unequal intensity of left and right circularly polarized light reaching to GaAs layer to excite unequal spin polarized carriers in GaAs layer, and (*iii*) (Ga,Mn)As as the spin filter layer for spin transport from GaAs to (Ga,Mn)As. Different from the previous experiments, the influence coming from the Zeeman splitting induced by an external magnetic field can be avoided here. While temperature dependence experiment indicates that the SPD-PC is mixed with the magnetic uncorrelated signals, which may come from current induced spin polarization.

Diluted magnetic semiconductors(DMS) have long been of great interest in combining the optical character of semiconductor and the magnetism character of ferromagnetic material^1^,^2^,^3^,^4^. (Ga,Mn)As is considered as one of the most promising DMS for spintronic devices due to the compatible growth techniques and relatively high Curie temperature^5^,^6^. As adding a new degree of freedom associated with spin, the optical properties of (Ga,Mn)As possess a circular dichroism. Relative phenomena, such as magnetico-optical(MO) effect^7^ and photoinduced magnetization^8^, have been widely observed. Because of the conservation of angular momentum, the circular dichroism can also be reflected by the polarization direction and the magnitude difference between the spin polarization carriers excited by left and right circularly polarized light. A further way to study magnetic properties of (Ga,Mn)As is to investigate the spin-polarized current or conductance^9^. Recently, spin-polarized photocurrents in DMS systems subjected to an external magnetic field induced by microwave or terahertz radiation have been observed, which is attributed to the giant Zeeman spin splitting or the spin-dependent carrier scattering^10^. So far almost all of the magnetic measurements, no matter the magnetic circular dichroism(MCD)^11^,^12^,^13^ or the spin-related current^9^,^10^, need an external magnetic field, which causes the Zeeman splitting inevitably and may make the results conflicting.

For DMS, the low spontaneous spin polarization degree especially at room temperature make it hard to be taken as a highly polarized spin injection source like metallic ferromagnetic material. While DMS overcome the dismatch between semiconductor and metal contact, the advantage of the interaction with the nonmagnetic semiconductor deserves more attention in the aspect of promoting spin injection efficiency and effective spin manipulation in nomagnetic semiconductors^14^. So the study on DMS/semiconductor heterostructure has more application value than the study on spontaneous spin polarization of (Ga,Mn)As itself. For example, it can be taken as the interface between ferromagnetic material and semiconductor to realize the carriers injection to nonmagnetic semiconductor which has been used to spin-led^15^,^16^. For the ferromagnetic/nonmagnetic multi-layer structure, such as (Ga,Mn)As/AlGaAs/(Ga,Mn)As^17^,^18^, the density of the carriers and the coherence between magnetic layers can be controlled by temperature, electric field *et al*., which can be used to the manufacture of magnetic or optical control superlattice devices.

In this work, we study the magnetic properties in the DMS (Ga,Mn)As/GaAs heterojunction by the photoconduction without a magnetic field. The obliquely incident circularly polarized light possesses in-plane angle momentum component, which directly interacts with the in plane spontaneous spin polarization of (Ga,Mn)As and avoids the influence of the giant Zeeman splitting induced by an external magnetic field. Different from the experiments that the substrate material is etched to investigate the photogenerated spin polarization in (Ga,Mn)As itself^13^, we remain the GaAs substrate to form a heterostructure to investigate the spin-polarized photoconduction, which may be related to the interaction of GaAs and (Ga,Mn)As including the interface effect and the spin filter effect of (Ga,Mn)As material when the optically generated carriers transport from GaAs to (Ga,Mn)As. We present the azimuth angle and the incident angle dependence of the spin polarization degree of photoconduction (SPD-PC), and find optical control of similar giant magneto resistance (GMR) effect. We also study the SPD-PC as a function of temperature, and find that the SPD-PC decreases with increasing temperature, and near or even above the Curie temperature, the SPD-PC still exists, which is beyond our expectation. We infer the SPD-PC contains non-magnetic related signals.

## Results

### Anisotropy study of the SPD-PC by rotating the incident light

**Figure 1:**
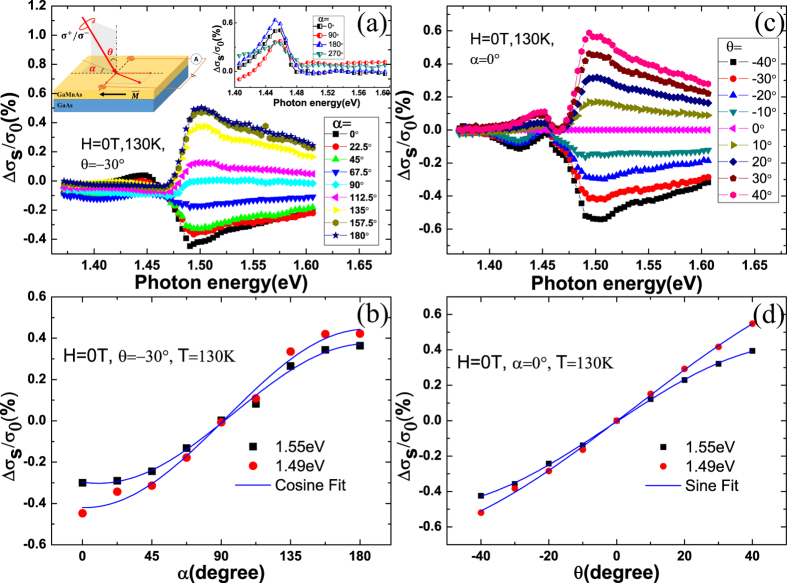
(**a**) Spectra of the SPD-PC detected in the (Ga,Mn)As/GaAs heterojunction with different azimuth angles ranged from 0° to 180°. The incident angle is fixed at 30°, and the temperature is 130 K. Inset on the top left corner is a schematic illustration of the geometry of the experiment. Inset on the top right corner is the contrast spectra of the SPD-PC detected in the GaAs bulk material with different azimuth angles. (**b**) The SPD-PC corresponding to 1.49 eV and 1.55 eV as a function of the azimuth angle in the (Ga,Mn)As/GaAs heterojunction. The solid line is the cosine fit. (**c**) Spectra of the SPD-PC detected in the (Ga,Mn)As/GaAs heterojunction with different incident angles ranged from −40° to 40°. The azimuth angle is fixed at 0°, and the temperature is 130 K. (**d**) The SPD-PC corresponding to 1.49 eV and 1.55 eV as a function of the incident angle in the (Ga,Mn)As/GaAs heterojunction. The solid line is the sine fit. Power of the excitation light for all experiments is 700 *mW*/*cm*^2^ at 1.55 eV, and no magnetic field is applied.

Since (Ga,Mn)As presents the in-plane magnetic anisotropy which determines the direction of magnetization when the temperature is lower than the Curie temperature, theoretically the absorption of left or right circularly polarized light along different crystal axes is different. As shown in the inset on the top left corner of Fig. 1(a), in the absent of the magnetic field, only the in plane component of the oblique incident circular polarized light can act on the magnetic moment, the in-plane angle momentum component along the magnetic easy axis is 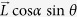. (*α* was the azimuth angle between the plane of the incident light and the direction of remnant magnetic moment direction 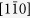, and *θ* was the incident angle). Assuming the photoconduction is proportional to the absorption, the SPD-PC should be expressed as Δ*σ*_*s*_/*σ*_0_ ∝ cos *α* sin *θ*.

We present first the SPD-PC on dependence of azimuth angle. Figure 1(a) shows the SPD-PC spectra with different *α* detected in (Ga,Mn)As/GaAs heterojunction. The incident angle *θ* is fixed at −30° and the experiment temperature is 130 K. We note that all of the spectra increase rapidly to a peak around 1.49~1.50 eV and then decreases gradually well beyond the peak value. Since the band gap energy *E*_*g*_ of (Ga,Mn)As is eventually very close to that of GaAs, both (Ga,Mn)As and GaAs can be excited in this energy range, so the observed SPD-PC peaks in Fig. 1(a) is the overlaps of the SPD-PC peaks of (Ga,Mn)As and GaAs. By varying *α*, the spectra present obvious anisotropy. Especially, after a 180° rotation, the sign of the SPD-PC is reversed. When *α* = 0° or 180°, the incident light irradiates along or against the magnetic moment, so the SPD-PC reaches up to the maximum or depresses to the minimum, which actually indicates that when the excitation light angle momentum component is consistent with the direction of magnetization, the SPD-PC is more prone to be generated. When *α* = 90°, Δ*σ*_*s*_/*σ*_0_ ≈ 0, this indicates that the interaction between the spin-polarized carriers and the magnetic moments vanishes when the incident plane is perpendicular to the direction of magnetization. In order to make sure the measured SPD-PC is closely related to the (Ga,Mn)As/GaAs structure, we also tested a GaAs sample without (Ga,Mn)As layer for contrast experiment, the experiment temperature is 210 K [see the inset on the top right corner of Fig. 1(a)]. For the GaAs sample, at four special angles of *α* = 0°, 90°, 180°, 270°, the SPD-PC does not change obviously let alone the sign conversion after a 180 degree rotation. This indicates that the observed cos *α* dependence of the SPD-PC on the azimuth angle is closely related to the spontaneous spin polarization of the (Ga,Mn)As layer or the interaction of the two layers. Figure 1(b) further shows *α* dependence of the SPD-PC stimulated by specific photon energy 1.49 eV and 1.55 eV, which is approximately equal to and greater than the *E*_*g*_ of (Ga,Mn)As. Δ*σ*_*s*_/*σ*_0_ varies as *cos* *α*, just as we think, the SPD-PC anisotropy is in accordance with the magnetic anisotropy.

We also measure the SPD-PC dependence on incident angle. Figure 1(c) shows the SPD-PC spectra with different *θ*, the azimuth angle is fixed at 0° and the experiment temperature is 130 K. When *θ* = 0°, there is no in-plane component of the incident light, as a result, the SPD-PC reach the minimum, with the increasement of *θ*, larger componet of the incident light is projected in plane and the SPD-PC also increases. The *θ* dependence of the SPD-PC can be seen more clearly in Fig. 1(d) corresponding to the exciting photoenergy of 1.49 eV and 1.55 eV. The SPD-PC changes as sin *θ*, just as we have expected. The sign conversion also happens when *θ* changing from *θ* to −*θ*, in fact this case is in accordance with the case when *α* is rotated by 180°.

### The SPD-PC varied with temperature

**Figure 2:**
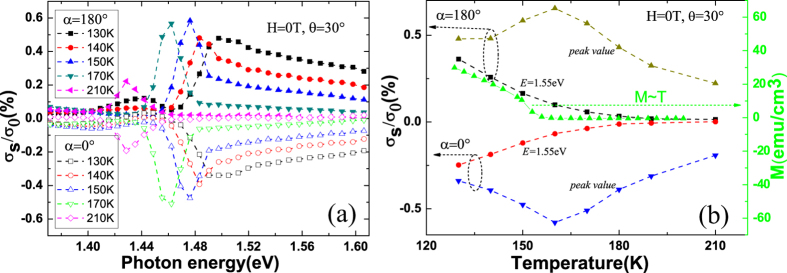
(**a**) Spectra of the SPD-PC detected in the (Ga,Mn)As/GaAs heterojunction with different temperatures ranged from 130 K to 210 K. The azimuth angle fixed at 0° or 180°, and the incident angle is fixed at 30°. Power of the excitation light was 700 *mW*/*cm*^2^, and no magnetic field is applied. (**b**) Temperature dependence of the SPD-PC corresponding to 1.55 eV (squares and circles) and the peak positions of the spectra (regular triangles and inverse triangles) detected in the (Ga,Mn)As/GaAs heterojunction with different azimuth angles. The green triangle line is the remnant magnetization on the dependence of temperature.

This behavior further studied in Fig. 2 shows the SPD-PC spectra with increasing temperatures from 130 to 210 K and the incident angle is fixed at 30°. As illustrated in Fig. 2, the peaks of the spectra show distinct blue shifts with the decreasing temperatures, and for *α* = 0° and *α* = 180° the spectra at each temperature have opposite sign even beyond the vicinity of the Curie temperature. We extract Δ*σ*_*s*_/*σ*_0_ from different temperatures at a fixed photon energy of 1.55 eV and the peak positions of the spectra from Fig. 2(a), as shown in Fig. 2(b). The SPD-PC at a fixed photon energy presents exponential decay as a function of temperature, while at the peak position the SPD-PC indeed doesn’t show monotonic increase with the decreasing temperature, but increases first up to 150 K then decreases. This is because the measured SPD-PC spectrum is not only associated with the magnetism, but also affected by the lifetime of the photo-generated carriers (*τ*), surface recombination rate (*ζ*_0_) and other factors. Their relationship with the photoconduction can be described as *σ* ∝ *τ*/*ζ*_0_^19^, *τ* and *ζ*_0_ are usually not constant values for different excitation energy and different temperatures. Such as for single spectrum at 150 K in Fig. 2(a), at band gap excitation, the photo-generated carriers have a longer lifetime, which contributes to the increase of the photoconduction, while when the excitation light energy continues increasing, the surface recombination rate is very large, which makes the photoconduction fell sharply. Besides, *τ* and *ζ*_0_ own different dependencies on temperature, so the fact that the SPD-PC value at the peak position doesn’t show monotonic increase with decreasing temperature is the competing result of the two parameters. Compared to the direct magnetic measurement *M* on temperature *T*, however, Δ*σ*_*s*_/*σ*_0_ has not disappeared above the Curie temperature whether at the peak position, or at a fixed energy. Moreover, the SPD-PC still has the cosine dependence on azimuth angle at this high temperature. This phenomenon is out of our expectation, which indicates that there exists the source independent of the magnetism.

## Discussion

From the above experimental results, we confirm that the SPD-PC can be generated by the oblique circularly polarized light in (Ga,Mn)As/GaAs heterojunction, and presents obvious in-plane anisotropy. We infer there may be three kinds of mechanisms to generate the SPD-PC in this system: (a) (Ga,Mn)As as the layer of photogenerated spin-polarized carriers, (b) GaAs as the layer of photogenerated spin-polarized carriers, (c) (Ga,Mn)As as the spin filter layer for spin transport from the GaAs to the (Ga,Mn)As.

**Figure 3:**
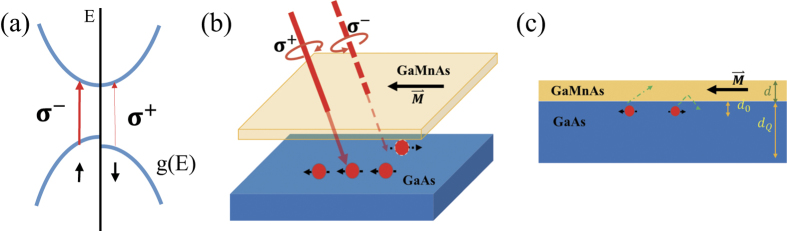
Schematic mechanisms to generate the SPD-PC. (**a**) Schematic distribution of the density of states for (Ga,Mn)As along 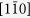 direction. As the valence band polarization, the concertration of spin-polarized carriers excited by left or right circularly polarized light is different according to the selection rule. (**b**) The SPD-PC generated in GaAs layer. Due to the absorption of (Ga,Mn)As, unequal intensity of left-handed light and right-handed light reaching to GaAs layer to generate unequal spin polarized carriers. (**c**) The SPD-PC generated through the interaction of the two layers. Spin polarized carriers in GaAs layer transport to (Ga,Mn)As layer, similar to GMR effect.

Compared to GaAs, the energy band of GaMnAs is modified by the interaction between holes and magneticions, whose Hamiltonian can be described as^20^ 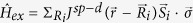, 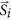 are the impurity spins at positions 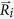, and 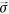 are the hole spins at position 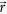, therefore the mean value of hole spins is not zero but parallel or antiparallel to magnetoionic spins, as shown in Fig. 3(a). As for our sample, the compressive strain due to the GaAs buffer makes the mean polarization of the holes stay in the plane^21^, the concertration of spin-polarized carriers excited by left or right circularly polarized light is different according to the selection rule. We take the excitation from heavy hole to conduction band as an example to analysis the mechanism of the SPD-PC. The difference of spin-polarized photoconductivity Δ*σ*_1_ between left-handed light and right-handed light coming from (Ga,Mn)As layer is 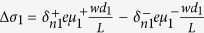, where *d*_1_ is the thickness of (Ga,Mn)As layer, *w, L* are the width, length of the sample, respectively. 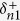 and 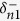 are the concentration excited by left-handed and right-handed polarized light in (Ga,Mn)As layer,respectively. 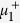 and 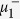 are the carriers mobility of spin up and spin down electrons in (Ga,Mn)As layer,respectively. We define the parameters independent with the polarized light as 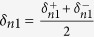, 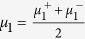 and dependent with the polarized light as 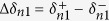, 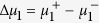. Considering (Ga,Mn)As as the layer of photogenerated spin-polarized carriers, the spin-polarized photoconductivity can be expressed as


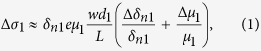


On the other hand, as the magnetic film is very thin, the laser may penetrate to the GaAs layer to generate carriers [see Fig. 3(b)]. For the GaAs layer itself, there is no net spin polarization in conduction band or valence band, therefore the transition probabilities for left and right circularly polarized light emission have no difference, even if we change the incident angle or azimuth angle [see the inset on the top right corner of Fig. 1(a)]. However, after the (Ga,Mn)As layer’s different absorption of left and right circularly polarized light, the intensity of the two polarized light reaching to the GaAs layer are also different, so the spin polarization of photogenerated carriers can also be generated in the GaAs layer. Considering GaAs as the layer of photogenerated spin-polarized carriers, the spin-polarized photoconductivity can be expressed as


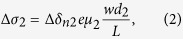


where *d*_2_ as the light absorption thickness of GaAs layer, *μ*_2_ are the photoinduced carriers mobility in GaAs, 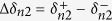, 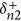 and 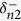 are the carriers concentration excited by left-handed and right-handed polarized light in GaAs layer, respectively. While by the driving of the bias, the photoinduced carriers in GaAs can go through the interface to (Ga,Mn)As and be collected by the external circuit. Due to the spin-dependent carrier scattering by localized magnetic ions, the (Ga,Mn)As layer shows an effect of spin filter for the polarized carriers from the GaAs layer, similar to GMR effect, that is the carriers in GaAs with spin orientation parallel to the remnant magnetization of (Ga,Mn)As, are easily transmitted through the high-conductivity spin channel while those with the antiparallel spin orientation are blocked at the interface [see Fig. 3(c)]. Considering (Ga,Mn)As as the spin filter layer, the spin-polarized photoconductivity can be expressed as


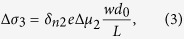


We define *d*_0_ as the effective interface thickness of GaAs influenced by (Ga,Mn)As layer, Δ*μ*_2_ the difference of carriers mobility excited by left-handed light and right-handed light at the interface of the GaAs layer. Now, Eqs 1, 2, 3 has describe the three types of mechanisms of the spin polarized photo conduction. The total polarization-independent photoconductivity can be expressed as 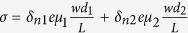, where the concentration of photoexcited carrier can be described by 
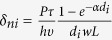
. Given the coefficient of light absorption *α* is 10^4^ *cm*^−1 ^22, *d*_1_ = 20 *nm* and *d*_2_ = 5 *μm*. Ignoring the difference of light power *P* and carrier lifetime *τ* in (Ga,Mn)As layer and GaAs layer, the commom photoconductivity ratio of (Ga,Mn)As layer and GaAs layer is estimated to be 
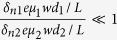
. So the total polarization-independent photoconductivity can be simplified to


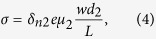


Overall, the SPD-PC of the three mechanisms can be expressed by
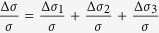



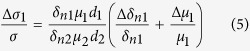



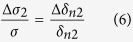



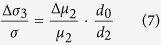


We can calculate that in Eq. 5 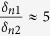. As GaAs bulk material has no selectivity for left or right circular polarized light, Δ*δ*_*n*2_ in Eq. 6 comes totally from the absorption difference of (Ga,Mn)As film directly, ignoring the spin relaxation difference between (Ga,Mn)As and GaAs, so we can assume 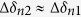. At present, the relevant experiments are basically carried out at very low temperatures, so we first discuss the dominance of these mechanisms at liquid helium temperature. We can get *μ*_1_ ≈ 2500 *cm*^2^/*V* · *s*^23^, *μ*_2_ ≈ 10^6^ *cm*^2^/*V* · *s* at 2 K^24^, and the effective interface thickness of GaAs *d*_0_ < 1 *nm*^25^. so Eqs 5, 6, 7 can be simplified as 
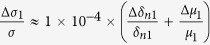
, 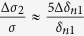, 
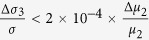
. According to the references, the absorption difference 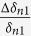 between left and right circularly polarized ligth is only about 2% at 2 K^13^, while the difference of carrier mobility between anti-parallel spin and parallel spin can be 3500 *cm*^2^/*V* · *s* (from the Fig. 2 in ref. 23)^23^. Substituting these parameters into Eqs 5, 6, 7, at low temperature we can get 
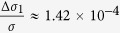
, 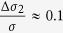, 
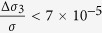
. Therefore, GaAs as the layer of photogenerated spin-polarized carriers makes a primary contribution to the SPD-PC. At higher low temperatures, the absorption difference and the carrier mobility difference will be reduced, but because of the great differences in the coefficient, the second mechanism that GaAs as the layer of photogenerated spin-polarized carriers may still be dominant.

**Figure 4:**
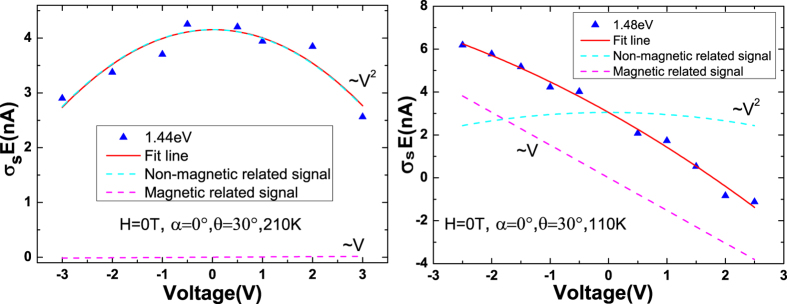
Voltage dependence of the spin photocurrent corresponding to (**a**) 1.44 eV at 210 K and (**b**) 1.48 eV at 110 K detected in the (Ga,Mn)As/GaAs heterojunction. The red solid line is the fit line, the blue dotted line is the square item of the fit 
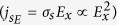
, which is non-magnetic related signal, and the purple dotted line is the linear item of the fit 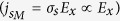, which is related to the magnetism.

**Figure 5:**
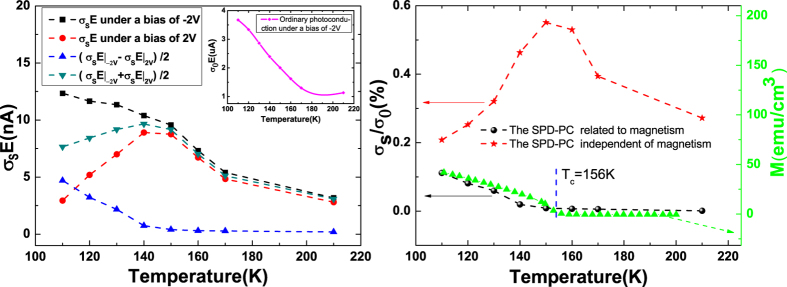
(**a**) Temperature dependence of the spin photocurrent (photoconduction) corresponding to the peak value detected in the (Ga,Mn)As/GaAs heterojunction with a bias of −2 V (black squares) and 2 V (red circles). The blue regular triangle line is the magnetism related photoconduction, calculated by 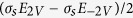. The green inverted triangle line is the CISP related photoconduction, calculated by 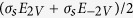. Inset on the top right corner is the ordinary photoconduction *σ*_0_ on the dependence of temperature under a bias of −2 V. (**b**) Temperature dependence of the SPD-PC corresponding to the peak value detected in the (Ga,Mn)As/GaAs heterojunction. The black sphere line and the red star line are the normalized results of the blue regular triangle line and the green inverted triangle line in Fig. 5(a) [calculated by 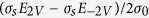 and 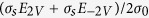], respectively. The green triangle line is the remnant magnetization on the dependence of temperature.

The temperature dependence of the SPD-PC spectra in Fig. 2 shows that the signal still exists even beyond the vicinity of the Curie temperature. If as we expected the SPD-PC is completely from the magnetic moment, the dependence on temperature should consist with the *M* ~ *T* curve. This phenomenon is out of our expectation, which indicates that there exists the source independent of the magnetism. In low-dimensional systems (quantum wells and single heterojunctions), an electric field leads to a stationary spin polarization of free charge carriers. The current-induced spin orientation in semiconductors had been widely studied^26^,^27^,^28^. J.I.Inoue proved that an electric field (*E*_*x*_) would suffice to induce a nonequilibrium magnetization or spin accumulation in the presence of the spin-orbit interaction, which can be expressed as 〈*S*_*y*_〉 = 4*πeτDλE*_*x*_, where *D* = *m*/2*πħ*^2^ is the density of state per spin, the lifetime *τ* is the momentum relaxation time and *λ* = *α*〈*E*_*z*_〉/*ħ* represents the Rashba interaction^29^. As the spin photoconduction excited by the left or right light is proportional to the carriers spin polarization 〈*S*_*y*_〉, the spin photocurrent should present the square relationship with the electrical field, that is 
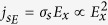
. While for the magnetism induced valence band polarization, the related spin photoconduction has nothing to do with the electrical field, so the spin photocurrent can be expressed as 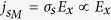. Theoretically, since the magnetism induced spin polarization above currier temperature almost disappears, 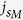 proportional to *E*_*x*_ inclines to zero, the measured signal is dominated by 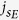 proportional to 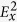. So the bias dependence of the signal above and below currier temperature is necessary, which help us to confirm the existence of 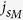 and 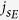. As shown in Fig. 4(a), the spin photocurrent corresponding to 1.44 eV at 210 K presents an obvious square dependence on voltage, which indicates the electrical field induced spin polarization is predominant when the temperature is above the Curie temperature. Similar experimental phenomena can also be observed in low-dimensional doped non-magnetic samples(see Supplementary Fig. S1 of the Supplementary Information). While in Fig. 4(b), an obvious linear voltage dependence of the spin photocurrent corresponding to 1.48 eV at 110 K is shown, which indicates an obvious magnetic signal below the Curie temperature. When reversing the bias, the spin photoconduction from the magnetism changes sign while from the CISP keeps unchanged. This provides us a method to extract the magnetism related 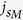 by the subtraction of the signals at V and −V, which can be expressed as 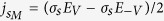, similarly the CISP related 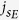 can be extracted by 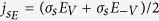. Figure 5(a) shows the temperature dependence of the spin photocurrent (photoconduction) corresponding to the peak value detected in the (Ga,Mn)As/GaAs heterojunction with a bias of −2 V (black squares, *τ*_*s*_*E*_−2*V*_) and 2 V (red circles, *τ*_*s*_*E*_2*V*_). The blue regular triangle line is 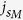 calculate by 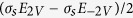. The green inverted triangle line is 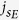 calculate by 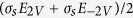. Figure 5(b) shows the temperature dependence of the SPD-PC corresponding to the peak value detected in the (Ga,Mn)As/GaAs heterojunction. The black sphere line and the red star line are the normalized results of the blue regular triangle line and the green inverted triangle line in Fig. 5(a) [calculated by 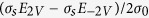 and 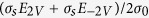], respectively. Consequently, we can remove the CISP related signals and obtain the magnetism related SPD-PC [the black sphere line in Fig. 5(b)]. Compared to the *M* ~ *T* curve, we find that the SPD-PC from the magnetism indeed vanishes beyond the Curie temperature [see the black sphere line in Fig. 5(b)]. In addition, we also find that the SPD-PC from the magnetism is quite different to that from the CISP [see the red star line in Fig. 5(b)]. The comparison between the two cases will be given elsewhere.

In conclusion, this work provides an optical way to generate the SPD-PC in (Ga,Mn)As/GaAs heterojunction without magnetic field. The spin polarized photocurrent controlled by optical and electric method realizes zero magnetic field spin manipulation which is the development direction of spintronics. The anisotropic magnetic related SPD-PC is attributed to three kinds of mechanisms, the first is self-producing due to valence band polarization of (Ga,Mn)As, the second is unequal intensity of left-handed light and right-handed light reaching to GaAs to generate unequal spin polarized carriers in GaAs layer, and the third is (Ga,Mn)As as the spin filter layer for spin transport from GaAs to (Ga,Mn)As. The SPD-PC demonstrates that the interface between (Ga,Mn)As and GaAs can realize effective spin injection and manipulation. While varying temperature experiment indicates that the SPD-PC is mixed with the magnetic uncorrelated signals, which may come from current induced spin polarization.

## Methods

### Sample preparation

Our *p*-type (Ga,Mn)As thin film has been deposited by molecular beam epitaxy at 200 °C onto a 100 nm undoped GaAs buffer layer grown on a semi-insulating GaAs (001) substrate. The Mn content and thickness of the magnetic layer are 8% and 20 nm, respectively. Compressive strain due to the GaAs buffer layer makes the (Ga,Mn)As layer result in the in-plane magnetic anisotropy^21^,^30^. By using magneto-transport and direct magnetization measurements, the (Ga,Mn)As sample shows obvious magnetic anisotropy and the magnetic easy axis is along 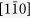 direction. The Curie temperature, from the magnetic measurement by a dc superconducting quantum interference device (SQUID) as a function of temperature, was determined to be 156 K. Besides, another sample of GaAs without (Ga,Mn)As layer was also prepared for contrast experiment.

### Measurement

The spin-polarized photoconduction were measured by applying a DC bias 3 V on the two contacts. A mode-locked Ti:sapphire laser goes through a polarizer and a photoelastic modulator(PEM), so the measured spin-polarized photoconduction is actually the photoconduction difference of (*σ*^+^) and (*σ*^−^). No magnetic field was applied and all the signals were collected by the lock-in amplifier. Meanwhile we also measured the polarization-independent photoconduction to monitor the total number of photoinduced carriers. The spin-polarized photoconduction is normalized by the polarization-independent photoconduction, which is called as spin polarization degree of photoconduction (SPD-PC).

## Additional Information

**How to cite this article**: Wu, Q. *et al*. Observation of spin-polarized photoconductivity in (Ga,Mn)As/GaAs heterojunction without magnetic field. *Sci. Rep.* **7**, 40558; doi: 10.1038/srep40558 (2017).

**Publisher's note:** Springer Nature remains neutral with regard to jurisdictional claims in published maps and institutional affiliations.

## Supplementary Material

Supplementary Information
