# Supplementary material for: Observation of spin-polarized photoconductivity in (Ga,Mn)As/GaAs heterojunction without magnetic field
Source: Sci Rep. 2017 Jan 13;7:40558. doi: 10.1038/srep40558 (PMC5233956; doi:10.1038/srep40558)
Supplement: Supplementary Information [file srep40558-s1.pdf]

Supplementary Material

Observation of spin-polarized photoconductivity in (Ga,Mn)As/GaAs heterojunction without magnetic field

Qing Wu[1], Yu Liu[1], Hailong Wang[2], Yuan Li[1], Wei Huang[1], Jianhua Zhao[2], and Yonghai Chen[1, *]

1 Key Laboratory of Semiconductor Materials Science, Beijing Key Laboratory of Low Dimensional Semiconductor Materials and Devices, Institute of Semiconductors, Chinese Academy of Sciences, and University of Chinese Academy of Sciences, Beijing, 100083, China
2 State Key Laboratory of Superlattices and Microstructures, Institute of Semiconductors, Chinese Academy of Sciences, and University of Chinese Academy of Sciences, Beijing, 100083, China
*yhchen@semi.ac.cn

We first conducted an experiment on undoped semi-insulating GaAs bulk materials by directly removing the GaMnAs layer, but didn't observe CISP related signals. Considering CISP may be related to impurity scattering that is absent in undoped GaAs, we added an experiment on Be doped p-type GaAs (non-magnetic doping) which is more similar to GaMnAs. We observed CISP related signals in it, as shown in Supplementary Figure S1, the measured signal had a square dependence on electrical field both at 120 K and 210 K ($j_s \propto E_x^2$), which was similar to that in GaMnAs/GaAs when magnetism disappeared [see Fig. 4(a) in the manuscript]. Since CISP has two main mechanisms, band spin-splitting and impurity-related spin scattering, we think the observed CISP related signal may be related to impurity-related spin scattering. The experiment on Be doped p-type GaAs helps us understand that CISP related signal also exists in non-magnetic sample, so the SPD-PC observed at the temperature higher than Tc of GaMnAs is not related to the magnetism of GaMnAs, but related to the impurity-related spin scattering of GaMnAs.

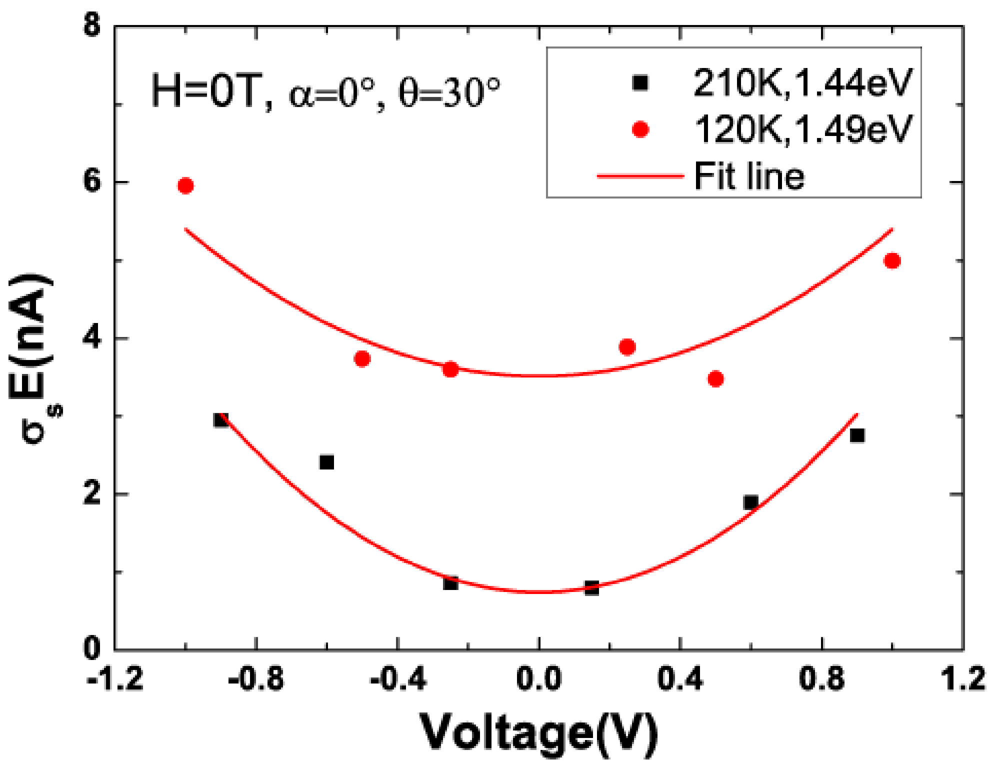

Supplementary Figure S1. Voltage dependence of the spin photocurrent corresponding to1.44 eV at 210 K (black squares) and 1.49eV at 120K (red circles) detected in Be doped p-type GaAs material. The red solid line is the fit line.
